# Effects of pentoxifylline on proteinuria and glucose control in patients with type 2 diabetes: a prospective randomized double-blind multicenter study

**DOI:** 10.1186/s13098-015-0060-1

**Published:** 2015-07-19

**Authors:** Seung Jin Han, Hae Jin Kim, Dae Jung Kim, Seung Soo Sheen, Choon Hee Chung, Chul Woo Ahn, Se Hwa Kim, Yong-Wook Cho, Seok Won Park, Soo-Kyung Kim, Chul Sik Kim, Kyung Wook Kim, Kwan Woo Lee

**Affiliations:** Department of Endocrinology and Metabolism, Ajou University School of Medicine, 164, World Cup-ro, Yeongtong-gu, Suwon, 443-380 Korea; Section of Clinical Epidemiology and Biostatistics in Clinical Trial Center, Ajou University School of Medicine, Suwon, 443-380 Korea; Department of Internal Medicine, Yonsei University Wonju College of Medicine, Wonju, 220-701 Korea; Department of Internal Medicine, Gangnam Severance Hospital, Yonsei University College of Medicine, Seoul, 135-720 Korea; Division of Endocrinology, Department of Internal Medicine, Catholic Kwandong University College of Medicine, Incheon, 404-834 Korea; Department of Internal Medicine, CHA Bundang Medical Center, CHA University, Seongnam, 463-712 Korea; Division of Endocrinology and Metabolism, Department of Internal Medicine, Hallym University Sacred Heart Hospital, Hallym University College of Medicine, Anyang, 431-796 Korea; Dongtan jeil Women’s Hospital, Hwaseong, 445-170 Korea; Severance Institute for Vascular and Metabolic Research, Yonesei University College of Medicine, Seoul, 120-752 Korea

**Keywords:** Pentoxifylline, Proteinuria, Insulin resistance, Diabetic nephropathy, Diabetes mellitus

## Abstract

**Background:**

Pentoxifylline is a methylxanthine derivative with significant anti-inflammatory, anti-fibrotic, and anti-proliferative properties. Studies have shown that pentoxifylline may have renoprotective effects in patients with diabetic nephropathy. However, most of these studies were limited by small sample sizes. Therefore, we investigated whether pentoxifylline could reduce proteinuria in patients with diabetic nephropathy and residual proteinuria who received an angiotensin-converting enzyme inhibitor (ACEI) or an angiotensin II receptor blocker (ARB). We also studied the effects of pentoxifylline on glycemic control, insulin resistance, and inflammatory parameters.

**Methods:**

This was a prospective, randomized double-blind, placebo-controlled, multi-center study. A total of 174 patients with type 2 diabetes and albuminuria (>30 mg/g of creatinine) who were taking the recommended dosage of ACEI or ARB for > 6 months and receiving conventional therapy for diabetes were randomly assigned to receive pentoxifylline (1200 mg, daily; n = 87) or a placebo (n = 87) for 6 months. The endpoints were the effects of pentoxifylline on proteinuria, renal function, glucose control, and inflammatory parameters.

**Results:**

The percentage changes in proteinuria from baseline in the pentoxifylline and placebo groups were a decrease of 23 % and 4 %, respectively (*p* = 0.012). In addition, significant reductions in fasting plasma glucose, glycated hemoglobin, and insulin resistance according to the homeostasis model assessment were observed in the pentoxifylline group compared to those in the placebo group. However there was no significant difference in serum tumor necrosis factor (TNF)-α between the groups.

**Conclusions:**

Pentoxifylline therapy reduced proteinuria and improved glucose control and insulin resistance without significant change of serum TNF-α in patients with type 2 diabetic nephropathy. Therefore, pentoxifylline is a potential therapeutic alternative for treating diabetes and diabetic nephropathy.

**Trial registration:**

NCT01382303

## Background

The incidence of diabetic nephropathy continues to increase worldwide in association with the sweeping rise in diabetes. In Korea, a rise in the number of patients with end-stage renal disease undergoing dialysis, which is the main contributor to the increasing prevalence of diabetic nephropathy, has been reported [1]. Thus, early detection and the effective management of diabetic nephropathy are needed to delay or prevent the progression of chronic kidney disease. Several factors are involved in the pathophysiology of diabetic nephropathy, including metabolic and hemodynamic alterations, oxidative stress, inflammation, and activation of the renin-angiotensin system (RAS) [2, 3]. Until now, blocking the RAS with an angiotensin-converting enzyme inhibitor (ACEI) or angiotensin II receptor blocker (ARB) was generally accepted as the standard treatment to delay the progression of diabetic nephropathy [4]. Although the treatment with ACEIs or ARBs decreases proteinuria, the risk of residual proteinuria remains; and these drugs do not stop the progression of diabetic nephropathy completely [5]. Therefore, many studies have been conducted using novel agents targeting their anti-inflammatory, anti-oxidant, anti-fibrotic, and intracellular modulatory properties [2].

The first use of pentoxifylline as a potential treatment for diabetic nephropathy occurred in the early 1980s [6]. Pentoxifylline is a non-selective phosphodiesterase inhibitor and adenosine receptor antagonist [7]. This methylxanthine derivative has been used primarily to treat peripheral vascular disease due to its hemorheologic and anti-inflammatory properties [8]. These properties are also associated with the pathogenesis of diabetic nephropathy; therefore, there has been interest in using pentoxifylline to treat diabetic nephropathy. Some studies reported that pentoxifylline reduced proteinuria in subjects with diabetes, while other studies have failed to show a clear-cut anti-proteinuric effect of pentoxifylline [9–15]. Another limitation is that most studies favoring pentoxifylline were poorly designed, with small sample sizes and flawed methodology, so evidence to support the use of pentoxifylline to treat diabetic nephropathy is insufficient [16].

In addition, studies examining the glycemic control effect of pentoxifylline have been reported intermittently since the 1970s [17–23]. Pentoxifylline exerts beneficial effects on obesity, glucose metabolism, and insulin resistance in patients with nonalcoholic fatty liver disease, which shares a common pathogenesis with diabetes, including insulin resistance, oxidative stress, and inflammation in recent studies [24, 25].

Therefore, we investigated whether pentoxifylline could reduce proteinuria in patients with diabetic nephropathy and residual proteinuria despite receiving an ACEI or ARB. We also studied the effects of pentoxifylline on glycemic control, insulin resistance, and inflammatory parameters.

## Methods

### Study subjects

In total, 174 subjects (103 males, 71 females; age 63.8 ± 9.6 years) were included in this study. Patients with type 2 diabetes who were ≥ 20 years old and who met the following criteria were eligible to participate: spot urinary albumin/creatinine (Cr) ratio > 30 mg/g on two consecutive measurements without other kidney or renal tract disease; blood pressure (BP) < 150/100 mmHg; treatment with an ACEI or ARB for > 6 months; glycated hemoglobin (HbA1c) < 10 %; and no drug changes within 4 weeks of randomization for this study.

The exclusion criteria included serum Cr > 2.0 mg/dL; a history of systemic inflammatory, immunological, or malignant diseases or cardiovascular disease in the previous 6 months; immunosuppressive or herb medication treatments; the use of pentoxifylline, cilostazol, ibudilast, or sildenafil citrate in the past 3 months; pregnancy, breastfeeding, or planning to become pregnant; and more than three-fold above the upper normal limits for aspartate aminotransferase (AST) and alanine aminotransferase (ALT).

### Study design

This was a prospective, randomized double-blind, placebo-controlled, multi-center study.

The study protocol was approved by the Institutional Review Board and ethics committee of the Ajou University School of Medicine (Suwon, Republic of Korea) and the Korean Ministry of Food and Drug Safety. This trial was registered with the US National Institutes of Health Clinical Trials (NCT01382303). All subjects gave informed consent. A total of 174 subjects were randomly assigned to either the pentoxifylline (Trental®, Han Dok Inc., Korea) (n = 87) or placebo group (n = 87) according to a computer-generated allocation system. Allocation was concealed by enclosing assignments in sequentially numbered, sealed opaque envelops, which were opened only after the enrolled subjects had completed all baseline assessments and treatment needed to be allocated.

Patients in the pentoxifylline group received 400 mg of pentoxifylline three times daily for 6 months, whereas the placebo group received identical starch tablets on the same schedule. The standard dose of pentoxifylline is 400 mg three times daily, which was used in previous studies that reported an anti-proteinuria effect of pentoxifylline [11, 26].

Patients were not allowed to change their medication during the study and were asked not to alter their current diet or physical activity. Participants were followed up after 3 and 6 months during the treatment period to evaluate the outcome measurements. Also, 1 month after the discontinuation of the study drug, urine samples were taken for measurement of proteinuria and albuminuria.

### Measurement of metabolic parameters

Body mass index (BMI) was calculated as weight (kg)/height squared (m^2^). Arterial BP was measured twice with a 5 min interval, using a standard mercury sphygmomanometer with the patient in a sitting position after a 5 min rest. The measurements were recorded at baseline and at the 3 and 6 months follow up visits.

Blood samples and single- first morning void urine samples were collected before breakfast in the morning after an 8–12-h overnight fast. The samples were collected in sterile tubes, centrifuged at 3000 × *g* for 10 min at 4 °C, and stored at –70 °C until analysis. Plasma glucose, insulin, HbA1c, serum Cr, AST, ALT, r-glutamyl transpeptidase(r-GT), serum high-sensitivity C-reactive protein (hs-CRP), serum and urine tumor necrosis factor (TNF)-α, and urinary protein, albumin and Cr levels were measured. All biochemical analyses were performed by Seoul Clinical Laboratories (Seoul, Republic of Korea).

Plasma glucose levels were measured with an automated enzymatic method. Insulin concentrations were measured with a microparticle enzyme immunoassay kit (Abbott, Mannheim, Germany) in subjects who were not receiving insulin treatment. Insulin resistance was evaluated according to the homeostatic model assessment insulin resistance (HOMA-IR) index ([fasting serum insulin (μIU/mL) × fasting serum glucose (mmol/l)]/22.5) [27]. The HbA1c concentration was determined by a turbidimetric inhibition immunoassay (Roche, Mannheim, Germany). Serum Cr and urine protein concentrations were measured using a standard colorimetric method [28]. Urine albumin concentrations were quantified by an immunoturbidimetric assay and urine Cr was measured using the Jaffe kinetic assay. The magnitude of urinary protein and albumin excretion was represented by the protein/Cr ratio and albumin/Cr ratio, respectively, in single-void urine samples [29]. The estimated glomerular filtration rate (eGFR) was determined by the simplified Modification of Diet in Renal Disease formula [30].

Serum AST and ALT levels were measured using the kinetic ultraviolet method according to the International Federation of Clinical Chemistry. Serum r-GT was measured using an enzymatic colorimetric assay with an automatic analyzer (Hitachi, Tokyo, Japan).

### Measurement of inflammatory parameters

Serum TNF-α concentrations were measured using a commercial available high-sensitivity enzyme-linked immunosorbent assay (ELISA) kit (R&D Systems, Minneapolis, MN, USA). The lower limit of detection is 0.106 pg/mL, and the intra- and inter-assay coefficients of variation of the assay were 3.1 and 7.2 %, respectively.

The urinary TNF-α values were below or just at the threshold of detection using a human TNF-α Platinum ELISA kit (eBioscience, San Diego, CA, USA); therefore, the values were measured again using a high-sensitivity ELISA kit (R&D Systems).

Serum hs-CRP levels were measured using a latex agglutination method and an automatic analyzer (Hitachi, Tokyo, Japan).

### Outcome measures

The primary end point was the percentage change from baseline to final on-treatment in proteinuria with pentoxifylline compared with the placebo group. Secondary end points included the percentage change of albuminuria and the mean change of eGFR, serum Cr, fasting glucose, HbA1c, HOMA-IR, hs-CRP, and serum TNF-α from baseline to final on-treatment.

### Statistical analyses

We calculated the necessary sample size based on a study of the combined effect of pentoxifylline and an ARB on proteinuria vs. an ARB only [31]. To detect a 25 % relative change with a Type 1 error rate of 0.05 and with a coefficient of variation of 0.55, the minimum required sample size for 90 % power was 69 in each group, and 87 after considering a 20 % drop-out rate.

All efficacy and safety analyses were conducted on the data in accordance with the intention-to-treat principle. Continuous variables are expressed as the mean ± standard deviation or median (interquartile range), and categorical variables are expressed as frequencies (percentages). The independent t-test or Mann–Whitney U-test was used to compare continuous variables between the groups according to the normality assumption. The χ2 test was implemented for categorical data, as appropriate.

The Mann–Whitney U-test was used to analyze the percentage change of proteinuria/albuminuria from baseline to final on-treatment between the placebo and the pentoxifylline groups. The remaining end points between the placebo and pentoxifylline groups were measured using the independent *t*-test. Changes within the groups were analyzed by paired *t*-tests at baseline and after 6 months of treatment.

Multivariate regression analysis was used to evaluate the independent association between pentoxifylline and the change of HbA1c. The correlation between changes in proteinuria and HbA1c from baseline to the end of the study was analyzed using Pearson correlation tests.

All statistical tests were two-tailed, and *p*-values < 0.05 were considered significant. All analyses were performed using SPSS for Windows ver. 19 (SPSS, Chicago, IL, USA).

## Results

**Fig. 1:**
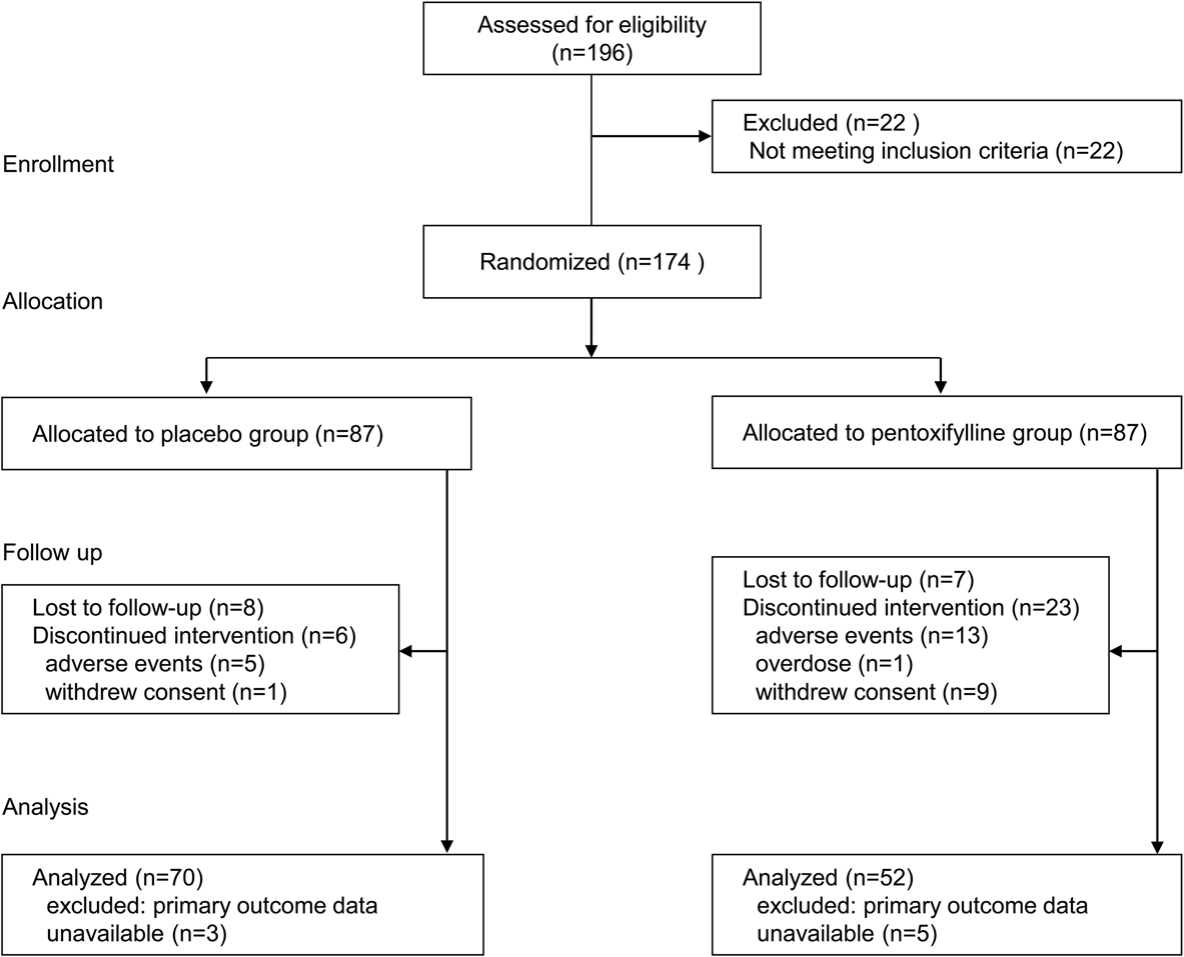
Flow diagram of the study

The disposition of the patients in the trial is shown in Fig. 1. A total of 174 of the 196 initially screened patients met the eligibility criteria and were randomly assigned to either the placebo or pentoxifylline group. Eight patients in the placebo and seven in the pentoxifylline group were lost to follow-up. Five subjects in the placebo group discontinued the study due to adverse events (AEs), and one subject withdrew for personal reasons. Thirteen subjects discontinued the study due to AEs in the pentoxifylline group; one subject was removed due to an overdose violation, and nine subjects withdrew for personal reasons. In total, 8 patients were excluded because urine data were unavailable and the 122 remaining were evaluated. The baseline demographic and clinical characteristics of the two groups were similar (Tables 1 and 2). No significant differences were observed in BP, renal function, urinary protein and albumin excretion, or metabolic parameters, except HbA1c. The pentoxifylline group had a higher HbA1c level than the placebo group (7.5 ± 0.9 vs. 7.2 ± 0.8 % (59 ± 10 vs. 56 ± 9 mmol/mol), *p* = 0.043). The urinary TNF-α concentration was below or just at the threshold of detection in all patients at baseline and remained so throughout the study (data not shown).

**Table 1:** Initial demographic parameters of the patients in the intention-to-treat population

	Placebo (n = 87)	Pentoxifylline (n = 87)	*P*-value
Age (years)	63.8 ± 9.4	63.7 ± 10.1	0.972
Sex (M/F)	56/31	47/40	0.217
Duration of diabetes (years)	12.6 ± 8.1	12.4 ± 8.3	0.917
BMI (kg/m^2^)	25.4 ± 3.5	26.0 ± 3.1	0.259
Systolic BP (mmHg)	127.8 ± 11.9	127.6 ± 14.1	0.938
Diastolic BP (mmHg)	74.0 ± 8.9	76.2 ± 10.4	0.141
Statin treatment (%)	56.3	50.0	0.448
Diabetes medication			
Sulfonylurea (%)	59.8	62.8	0.755
Metformin (%)	83.9	86.0	0.832
Thiazolidinedione (%)	5.7	7.0	0.766
DPP-4 inhibitor (%)	25.3	25.6	1.000
Insulin (%)	13.8	16.3	0.676

Data are presented as means ± standard deviation (SD), or numbers (percentage)

*BMI* body mass index, *BP* blood pressure, *DPP-4* dipeptidyl peptidase 4

The independent *t*-test was used to compare continuous variables between the groups. The χ2 test was implemented for categorical data

**Table 2:** Initial biochemical parameters of the patients in the intention-to-treat population

	Placebo (n = 87)	Pentoxifylline (n = 87)	*P*-value
Serum Cr (mg/dL)	0.9 ± 0.3	0.9 ± 0.2	0.165
Urinary Cr (mg/dL)	103 (72-136)	86 (61-122)	0.176
eGFR (ml/min per 1.73 m^2^)	85.4 ± 27.0	88.8 ± 24.8	0.401
Proteinuria (mg/g)	395 (229–714)	371 (218–610)	0.689
Albuminuria (mg/g)	203 (88-513)	143 (71-315)	0.201
Fasting plasma glucose (mg/dL)	140 ± 35	138 ± 35	0.795
HbA1c (%)	7.2 ± 0.8	7.5 ± 0.9	0.043
AST(U/L)	26.5 ± 14.9	24.8 ± 8.7	0.368
ALT(U/L)	26.5 ± 18.0	26.3 ± 12.4	0.910
r-GT(U/L)	39.0 ± 36.2	35.4 ± 32.9	0.515
hs-CRP (mg/L)	1.7 ± 2.3	1.8 ± 2.8	0.667
Serum TNF-α (pg/mL)	1.4 ± 1.4	1.6 ± 2.8	0.570

Data are presented as means ± standard deviation (SD) or medians (interquartile ranges)

*Cr* creatinine, *eGFR* estimated glomerular filtration rate, *HbA1c* glycated hemoglobin, *AST* aspartate aminotransferase, *ALT* alanine aminotransferase, *r-GT* r-glutamyl transpeptidase, *hs-CRP* high-sensitivity C-reactive protein, *TNF* tumor necrosis factor

The independent *t*-test or Mann–Whitney U-test was used to compare continuous variables between the groups according to the normality assumption

**Fig. 2:**
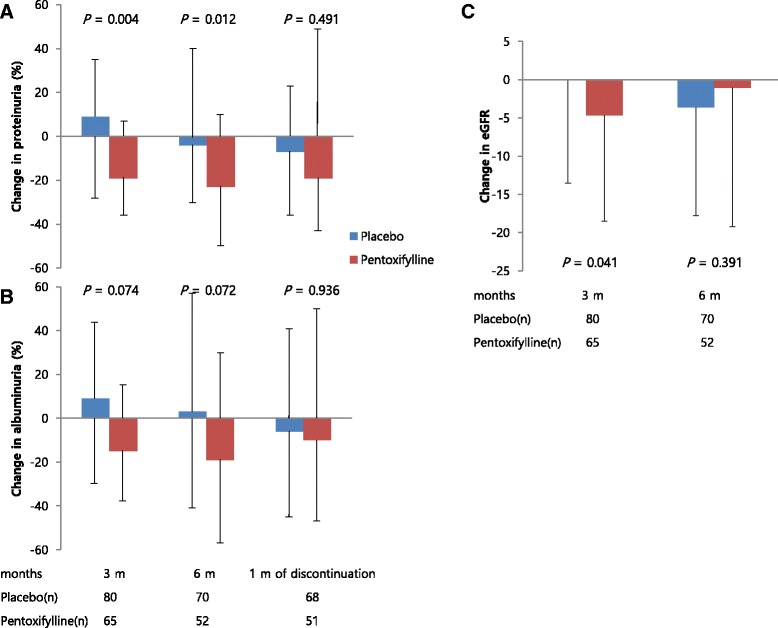
Changes in proteinuria and renal function induced by placebo or pentoxifylline. **a** Proteinuria change was the percentage change from baseline and expressed as median with interquartile range. **b** Albuminuria change was the percentage change from baseline and expressed as median with interquartile range. **c** The eGFR change was the change from baseline and expressed as mean ± standard deviation (SD). eGFR, estimated glomerular filtration rate. *P*-values are for comparisons of each variable between the placebo and pentoxifylline groups at the corresponding time points

At the 3- and 6-month treatment time points, proteinuria in the pentoxifylline group decreased significantly compared to that in the placebo group (3 months: 19 % decrease (−36 to 7) vs. 9 % increase (−28 to 35), *p* = 0.004; 6 months: 23 % decrease (−50 to 10) vs. 4 % decrease (−30 to 40), *p* = 0.012) (Fig. 2). The pentoxifylline group showed a trend toward decreased albuminuria (3 months: 15 % decrease (−38 to 15) vs. 9 % increase (−30 to 44), *p* = 0.074; 6 months: 19 % decrease (−57 to 30) vs. 3 % increase (−41 to 57), *p* = 0.072).

Subgroup analyses were performed to determine whether baseline proteinuria and HbA1c influenced any change in proteinuria. In the subjects with microalbuminuria (n = 87), pentoxifylline group showed a significant decrease in proteinuria and albuminuria compared to those in the placebo group (proteinuria: 26 % decrease (−47 to 9) vs. 4 % decrease (−25 to 30), *p* = 0.012; albuminuria: 36 % decrease (−58 to 33) vs. 12 % increase (−41 to 93), *p* = 0.029). However, no significant differences were observed between the groups in the subjects with macroalbuminuria (n = 35, proteinuria: 23 % decrease (−54 to 21) vs. 5 % decrease (−43 to 44), *p* = 0.405; albuminuria: 18 % decrease (−40 to 29) vs. 20 % decrease (−46 to 50), *p* = 0.947). In addition, there was no significant difference in primary outcome between the groups according to baseline median HbA1c levels (HbA1c ≤ 7.175 (n = 61), 29 % decrease (−52 to 8) vs. 5 % decrease (−30 to 44), *p* = 0.112; HbA1c >7.175 (n = 61), 22 % decrease (−47 to 17) vs. 2 % decrease (−33 to 28), *p* = 0.106).

To evaluate whether long term anti-proteinuric effects of pentoxifylline remained 1 month after discontinuation of treatment, we collected urine samples. No significant change in proteinuria and albuminuria was observed between the groups (proteinuria: pentoxifylline vs. placebo 19 % decrease (−43 to 49) vs. 7 % decrease (−36 to 23), *p* = 0.491; albuminuria 10 % decrease (−47 to 50) vs. 6 % decrease (−45 to 41), *p* = 0.936, Fig. 2).

**Fig. 3:**
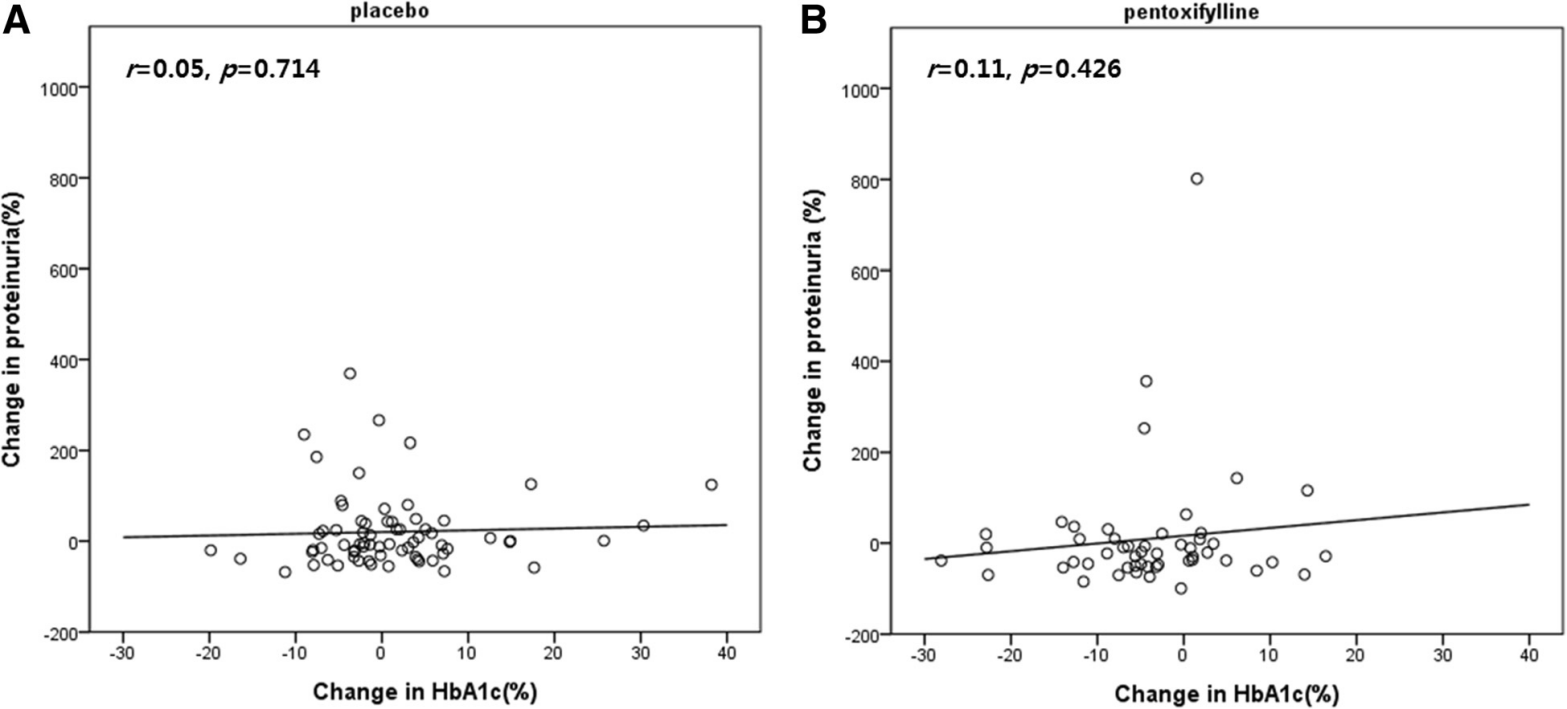
The relationship between the change in proteinuria and HbA1c from baseline. **a** placebo group, **b** pentoxifylline group

Although eGFR in the pentoxifylline group declined significantly after 3 months of treatment compared to that of the placebo group, there were no significant differences between the two groups after 6 months of treatment (eGFR: −1.05 ± 18.20 vs. −3.59 ± 14.20, *p* = 0.391, Fig. 2). In addition, no significant changes in the serum Cr level were observed between the groups (pentoxifylline vs. placebo 0.03 ± 0.16 vs. 0.04 ± 0.15, *p* = 0.801). The changes in BMI, systolic BP, and diastolic BP did not differ significantly between the two groups (Table 3). Treatment with pentoxifylline significantly reduced the HbA1c level, fasting glucose level, and HOMA-IR value compared to those in the placebo group (*p* < 0.05). HbA1c decreased significantly in the pentoxifylline group (from 7.6 ± 0.9 to 7.2 ± 0.9 % (59 ± 10 to 55 ± 10 mmol/mol), *p* < 0.05), without a significant change in the placebo group. To confirm the glucose-lowering effect of pentoxifylline, we performed multivariate regression analysis with the change in HbA1c as the dependent variable and clinical parameters as independent variables. This indicated that pentoxifylline treatment was an independent factor associated with a change of HbA1c after adjusting for baseline HbA1c, age, sex, change in BMI, and duration of diabetes (*p* = 0.003). Figure 3 shows the relationship between the changes in proteinuria and HbA1c from baseline to 6 months of treatment. There was no significant correlation within either group (Fig. 3). No significant changes in hs-CRP, serum TNF-α, AST, ALT, r-GT were observed in either group during the study.

**Table 3:** Changes in metabolic parameters induced by the placebo or pentoxifylline at the end of treatment

	Placebo (n = 70)	Pentoxifylline (n = 52)	*P*-value
BMI (kg/m^2^)	0.22 ± 1.75	−0.25 ± 0.80	0.079
Systolic BP (mmHg)	3.9 ± 18.3	1.3 ± 15.7	0.417
Diastolic BP (mmHg)	0.3 ± 11.8	1.8 ± 10.9	0.473
HbA1c (%)	0.09 ± 0.72	−0.34 ± 0.74	0.002
Fasting plasma glucose (mg/dL)	8.4 ± 37.4	−10.0 ± 38.9	0.009
HOMA-IR^a^	0.24 ± 2.61	−0.79 ± 2.03	0.041
Hs-CRP (mg/L)	0.70 ± 4.33	0.51 ± 4.32	0.818
Serum TNF-α (pg/mL)	0.14 ± 0.71	−0.24 ± 3.11	0.322
AST(U/L)	0.33 ± 13.33	−1.31 ± 7.84	0.432
ALT(U/L)	−0.30 ± 14.38	−1.37 ± 8.75	0.637
r-GT(U/L)	1.19 ± 25.27	−3.90 ± 8.88	0.126

Data expressed as the means ± standard deviation (SD). *P*-values are for comparing the absolute change in each variable between the placebo and pentoxifylline groups

^a^HOMA-IR was measured only in subjects who were not receiving insulin treatment, placebo (n = 58), pentoxifylline (n = 39)

*BMI* body mass index, *BP* blood pressure, *HbA1c* glycated hemoglobin, *HOMA-IR* homeostatic model assessment insulin resistance, *hs-CRP* high-sensitivity C-reactive protein, *TNF* tumor necrosis factor, *AST* aspartate aminotransferase, *ALT* alanine aminotransferase, *r-GT*, r-glutamyl transpeptidase

The frequency of AEs was higher in the pentoxifylline group than in the placebo group (Table 4). No drug-related serious AEs were reported. The major AEs in the pentoxifylline group were gastrointestinal disturbances such as dyspepsia, nausea, vomiting, gastric reflux, diarrhea, constipation and headache. Drug-related or non-drug-related AEs led to discontinuing the medication in 18 patients: five in the placebo group (femoral fracture, lumbar spine fracture, acute hepatitis, nausea, and hyperglycemia with nausea) and 13 in the pentoxifylline group (ten with gastrointestinal disturbances, headache, dizziness, and flushing).

**Table 4:** Overall summary of patients with an adverse event

Adverse events	Placebo (n = 87)	Pentoxifylline (n = 87)
Overall summary of patients with an AE, n (%)		
One or more AE, n (%)	20 (23.0)	27 (31.0)
One or more drug-related AE, n (%)	12 (13.8)	23 (26.4)
AE leading to discontinuation	5 (5.7)	13 (14.9)
Patients with AEs of ≥3 % in any group, n (%)		
Dyspepsia	1 (1.1)	7 (8.0)
Nausea/vomiting	2 (2.3)	5 (5.7)
Gastric reflux	3 (3.4)	5 (5.7)
Diarrhea	0 (0.0)	2 (2.3)
Constipation	0 (0.0)	1 (1.1)
Headache	1 (1.1)	3 (3.4)

*AE* adverse event

## Discussion

Proteinuria is an important predictor of end stage renal disease in patients with diabetic nephropathy [32]. Reducing proteinuria is effective in delaying the progression of chronic renal disease, as well as decreasing cardiovascular morbidity and mortality [33, 34].

We found that a 6-month pentoxifylline treatment added to a RAS inhibitor reduced proteinuria, the HbA1c level, and the HOMA-IR value compared with placebo. No significant relationship was observed between the changes of proteinuria and the change in HbA1c. Therefore, the additive anti-proteinuric effect of pentoxifylline was independent of improved glycemic control. Our results confirm reports of an anti-proteinuric effect of pentoxifylline in patients with diabetic nephropathy and show the beneficial effects of maintaining glucose homeostasis [11, 12, 35].

Pentoxifylline treatment showed a trend of reducing albuminuria, however displayed significant reductions of both proteinuria and albuminuria in microalbuminuria subgroup compared to those in the placebo group. Therefore, we think heterogeneity in subjects’ characteristics influences the results of albuminuria. Interestingly, our results are contrary to a meta-analysis by McCormick et al. (2008), which showed that pentoxifylline decreased proteinuria in patients with macroalbuminuria, but not with microalbuminuria [9]. However, our study consisted of a small proportion of subjects with macroalbuminuria, which could be the reason for the differences compared with the previous study.

Whether the additive anti-proteinuric effect of pentoxifylline remains after discontinuation has not been demonstrated to date. We found that the anti-proteinuric effect of pentoxifylline was not sustained 1 month after drug discontinuation.

No significant difference in eGFR was observed between the groups. Our trial included subjects with predominantly preserved renal function (serum Cr ≤ 2.0 mg/dL). By contrast, most studies showing beneficial effects of pentoxifylline on eGFR enrolled mostly patients with advanced chronic kidney disease [13, 15, 35]. An investigation of the effect of pentoxifylline on the eGFR in lower-risk patients will require a longer follow-up period to observe differences. In addition, there was no significant difference in serum Cr between the groups. Although Rodriguez-Moran et al. (2006) reported that pentoxifylline significantly reduced serum Cr levels, a recent meta-analysis of six randomized controlled trials showed results similar to ours; i.e., that combining pentoxifylline with RAS inhibitors did not significantly change the serum Cr levels [36, 37].

The spot urine protein/Cr ratio has been used in place of the 24-h urine protein collection test on the basis of the assumption that the urine protein excretion rate, which is proportional to the urine Cr excretion rate, is relatively constant throughout the day [29]. There was no significant difference in urinary Cr levels at baseline and after treatment between the two groups.

The mechanisms underlying the anti-proteinuric effects of pentoxifylline remain unknown, although some possibilities can be discussed. The first is antagonist action on adenosine 2 receptors that modulate GFR and the renal action of atrial natriuretic factor [10, 33]. Second, the hemorheologic effect of pentoxifylline promotes beneficial changes in blood flow by improving blood fluidity in the peritubular plexus and reducing overload of low-molecular-weight proteins into the proximal tubule, actions that reduce intra-glomerular pressure [34]. Finally, pentoxifylline decreases intrarenal inflammation based on its anti-TNF-α properties [35, 38].

Contrary to our expectations, we failed to observe any change in serum TNF-α associated with pentoxifylline treatment. Similarly, pentoxifylline failed to significantly reduce serum TNF-α levels in patients with chronic kidney disease [38]. A meta-analysis of pentoxifylline for treating nonalcoholic fatty liver disease also failed to show changes in serum levels [24]. The reasons for this inconsistent result are unclear; however, the lack of change in circulating TNF-α might not correlate with tissue levels.

We noted beneficial effects of pentoxifylline on glucose control and insulin resistance (HOMA-IR). Earlier studies of the effects of pentoxifylline on glucose metabolism showed glucose-lowering effects by stimulating insulin secretion by increasing intracellular cAMP levels [17, 19]. Pentoxifylline potentiates amino acid- and glibenclamide-induced insulin secretion [17, 18]. Raptis *et al*. (1987) reported that 14 days of the daily administration of 1200 mg of pentoxifylline led to a decrease in blood glucose and a smoothing of glucose fluctuations in patients with type 2 diabetes [19]. They suggested that pentoxifylline augmented insulin secretion and improved peripheral glucose utilization. In another study, administering pentoxifylline transiently inhibited endogenous glucose production in healthy subjects [20].

Pentoxifylline has anti-inflammatory and anti-oxidant abilities, which play important roles in the pathogenesis of diabetes [22, 23, 39, 40]. Studies have shown that pentoxifylline improves glucose control by downregulating the pro-inflammatory cytokine-mediated nitric oxide synthase pathway in an animal model of diabetes [21, 22]. Garcia *et al*. reported that pentoxifylline has glucose lowering effects, at least partly related to the inhibition of ATP-sensitive K+ and suppression of TNF-α, inducible nitric oxide synthase and cyclooxygenase-2 in the pancreas of diabetic rats [23].

Recently, a few studies reported the effects of pentoxifylline on glucose and insulin resistance in patients with nonalcoholic fatty liver disease, which has a somewhat similar pathogenesis as diabetic nephropathy. In these studies, 6 months of pentoxifylline therapy showed a significant reduction in HOMA-IR as well as in liver enzymes [41, 42]. In a meta-analysis of randomized double-blind placebo-controlled studies, pentoxifylline treatment led to a significant reduction in glucose [24]. The underlying mechanism for the glucose-lowering effect of pentoxifylline needs to be investigated in future studies.

Although our study was well-designed, it has limitations. First, there was an unexpectedly high rate of AEs in our study due to pentoxifylline treatment, which limited adherence to the therapy. Gastrointestinal disturbances are well-recognized potential AEs of pentoxifylline. These effects are dose-related and dosage form-related, with a reported higher frequency in patients taking a higher dosage and in those taking an immediate-release form compared to those taking an extended-release form. Because our subjects could not tolerate doses of 1200 mg/day of immediate-release pentoxifylline compared to other study populations, we hypothesize that dosage up-titration with an extended-release form may decrease the rate of AEs [35, 43]. Although the unexpectedly high dropout rate in the pentoxifylline group meant that the study did not reach the desired statistical power, the effects of pentoxifylline on proteinuria were still positive. Second, although the patients were asked not to alter their current diet and physical activity, we did not evaluate the potential role of these factors in the results. Third, the follow-up period was relatively short. Finally, as we used only a single first morning void urine sample, instead of three consecutive first morning void urine samples, the measurements of proteinuria may be less accurate.

## Conclusions

In conclusion, adding pentoxifylline to an ACEI or ARB reduced proteinuria in Korean patients with type 2 diabetic nephropathy and exerted beneficial effects on glucose control and insulin resistance. Therefore, pentoxifylline is a potential therapeutic alternative for treating diabetes and diabetic nephropathy.

## References

[1] Park CW (2014). Diabetic kidney disease: from epidemiology to clinical perspectives. Diabetes Metab J.

[2] Mima A, Qi W, King GL (2012). Implications of treatment that target protective mechanisms against diabetic nephropathy. Semin Nephrol.

[3] Navarro-Gonzalez JF, Mora-Fernandez C (2008). The role of inflammatory cytokines in diabetic nephropathy. J Am Soc Nephrol.

[4] American Diabetes, Association (2014). Standards of medical care in diabetes--2014. Diabetes Care.

[5] Nobakht N, Kamgar M, Rastogi A, Schrier RW (2011). Limitations of angiotensin inhibition. Nat Rev Nephrol.

[6] Blagosklonnaia Ia V, Mamedov R, Kozlov VV, Emanuel VL, Kudriashova MI (1982). Effect of trental on indices kidney function in diabetes mellitus. Probl Endokrinol (Mosk).

[7] Windmeier C, Gressner AM (1997). Pharmacological aspects of pentoxifylline with emphasis on its inhibitory actions on hepatic fibrogenesis. Gen Pharmacol.

[8] Ward A, Clissold SP (1987). Pentoxifylline. A review of its pharmacodynamic and pharmacokinetic properties, and its therapeutic efficacy. Drugs.

[9] McCormick BB, Sydor A, Akbari A, Fergusson D, Doucette S, Knoll G (2008). The effect of pentoxifylline on proteinuria in diabetic kidney disease: a meta-analysis. Am J Kidney Dis.

[10] Rodriguez-Moran M, Guerrero-Romero F (2008). Efficacy of pentoxifylline in the management of microalbuminuria in patients with diabetes. Curr Diabetes Rev.

[11] Navarro JF, Mora C, Muros M, Garcia J (2005). Additive antiproteinuric effect of pentoxifylline in patients with type 2 diabetes under angiotensin II receptor blockade: a short-term, randomized, controlled trial. J Am Soc Nephrol.

[12] Navarro JF, Mora C, Muros M, Maca M, Garca J (2003). Effects of pentoxifylline administration on urinary N-acetyl-beta-glucosaminidase excretion in type 2 diabetic patients: a short-term, prospective, randomized study. Am J Kidney Dis.

[13] Perkins RM, Aboudara MC, Uy AL, Olson SW, Cushner HM, Yuan CM (2009). Effect of pentoxifylline on GFR decline in CKD: a pilot, double-blind, randomized, placebo-controlled trial. Am J Kidney Dis.

[14] Diskin CJ, Stokes TJ, Dansby LM, Radcliff L, Carter TB (2007). Will the addition of pentoxifylline reduce proteinuria in patients with diabetic glomerulosclerosis refractory to maximal doses of both an angiotensin-converting enzyme inhibitor and an angiotensin receptor blocker?. J Nephrol.

[15] Goicoechea M, Garcia De Vinuesa S, Quiroga B, Verdalles U, Barraca D, Yuste C (2012). Effects of pentoxifylline on inflammatory parameters in chronic kidney disease patients: a randomized trial. J Nephrol.

[16] Shan D, Wu HM, Yuan QY, Li J, Zhou RL, Liu GJ (2012). Pentoxifylline for diabetic kidney disease. Cochrane Database Syst Rev.

[17] Raptis S, Pfeiffer EF (1972). Progress in oral therapy of diabetes mellitus with sulphonylureas of the second generation. Acta Diabetol Lat.

[18] Basabe JC, Udrisar DP, Knopf CF, Aparicio N (1977). The influence of pentoxyfylline [1-(5-oxohexyl-) 3,7-dimethylxanthine] (BL 191) on the insulin secretion induced by glibenclamide and by arginine/glucose in the perfused pancreas. Acta Diabetol Lat.

[19] Raptis S, Mitrakou A, Hadjidakis D, Diamantopoulos E, Anastasiou C, Fountas A (1987). 24-h blood glucose pattern in type I and type II diabetics after oral treatment with pentoxifylline as assessed by artificial endocrine pancreas. Acta Diabetol Lat.

[20] Corssmit EP, Romijn JA, Endert E, Sauerwein HP (1994). Pentoxifylline inhibits basal glucose production in humans. J Appl Physiol (1985).

[21] Stosic-Grujicic SD, Maksimovic DD, Stojkovic MB, Lukic ML (2001). Pentoxifylline prevents autoimmune mediated inflammation in low dose streptozotocin induced diabetes. Dev Immunol.

[22] Stosic-Grujicic S, Maksimovic D, Badovinac V, Samardzic T, Trajkovic V, Lukic M (2001). Antidiabetogenic effect of pentoxifylline is associated with systemic and target tissue modulation of cytokines and nitric oxide production. J Autoimmun.

[23] Garcia FA, Pinto SF, Cavalcante AF, Lucetti LT, Menezes SM, Felipe CF (2014). Pentoxifylline decreases glycemia levels and TNF-alpha, iNOS and COX-2 expressions in diabetic rat pancreas. Springerplus.

[24] Zeng T, Zhang CL, Zhao XL, Xie KQ (2014). Pentoxifylline for the treatment of nonalcoholic fatty liver disease: a meta-analysis of randomized double-blind, placebo-controlled studies. Eur J Gastroenterol Hepatol.

[25] Du J, Ma YY, Yu CH, Li YM (2014). Effects of pentoxifylline on nonalcoholic fatty liver disease: a meta-analysis. World J Gastroenterol.

[26] Rodriguez-Moran M, Guerrero-Romero F (2005). Pentoxifylline is as effective as captopril in the reduction of microalbuminuria in non-hypertensive type 2 diabetic patients--a randomized, equivalent trial. Clin Nephrol.

[27] Bonora E, Targher G, Alberiche M, Bonadonna RC, Saggiani F, Zenere MB (2000). Homeostasis model assessment closely mirrors the glucose clamp technique in the assessment of insulin sensitivity: studies in subjects with various degrees of glucose tolerance and insulin sensitivity. Diabetes Care.

[28] Yatzidis H (1977). New colorimetric method for quantitative determination of protein in urine. Clin Chem.

[29] Schwab SJ, Christensen RL, Dougherty K, Klahr S (1987). Quantitation of proteinuria by the use of protein-to-creatinine ratios in single urine samples. Arch Intern Med.

[30] Levey AS, Bosch JP, Lewis JB, Greene T, Rogers N, Roth D (1999). A more accurate method to estimate glomerular filtration rate from serum creatinine: a new prediction equation. Modification of Diet in Renal Disease Study Group. Ann Intern Med.

[31] Lin SL, Chen YM, Chiang WC, Wu KD, Tsai TJ (2008). Effect of pentoxifylline in addition to losartan on proteinuria and GFR in CKD: a 12-month randomized trial. Am J Kidney Dis.

[32] Zhang Z, Shahinfar S, Keane WF, Ramjit D, Dickson TZ, Gleim GW (2005). Importance of baseline distribution of proteinuria in renal outcomes trials: lessons from the reduction of endpoints in NIDDM with the angiotensin II antagonist losartan (RENAAL) study. J Am Soc Nephrol.

[33] Eijkelkamp WB, Zhang Z, Remuzzi G, Parving HH, Cooper ME, Keane WF (2007). Albuminuria is a target for renoprotective therapy independent from blood pressure in patients with type 2 diabetic nephropathy: post hoc analysis from the Reduction of Endpoints in NIDDM with the Angiotensin II Antagonist Losartan (RENAAL) trial. J Am Soc Nephrol.

[34] Olsen MH, Wachtell K, Ibsen H, Lindholm LH, Dahlof B, Devereux RB (2006). Reductions in albuminuria and in electrocardiographic left ventricular hypertrophy independently improve prognosis in hypertension: the LIFE study. J Hypertens.

[35] Navarro-Gonzalez JF, Mora-Fernandez C, Muros de Fuentes M, Chahin J, Mendez ML, Gallego E (2015). Effect of Pentoxifylline on Renal Function and Urinary Albumin Excretion in Patients with Diabetic Kidney Disease: The PREDIAN Trial. J Am Soc Nephrol.

[36] Rodriguez-Moran M, Gonzalez-Gonzalez G, Bermudez-Barba MV, Medina de la Garza CE, Tamez-Perez HE, Martinez-Martinez FJ (2006). Effects of pentoxifylline on the urinary protein excretion profile of type 2 diabetic patients with microproteinuria: a double-blind, placebo-controlled randomized trial. Clin Nephrol.

[37] Tian ML, Shen Y, Sun ZL, Zha Y (2015). Efficacy and safety of combining pentoxifylline with angiotensin-converting enzyme inhibitor or angiotensin II receptor blocker in diabetic nephropathy: a meta-analysis. Int Urol Nephrol.

[38] Chen YM, Lin SL, Chiang WC, Wu KD, Tsai TJ (2006). Pentoxifylline ameliorates proteinuria through suppression of renal monocyte chemoattractant protein-1 in patients with proteinuric primary glomerular diseases. Kidney Int.

[39] Radfar M, Larijani B, Hadjibabaie M, Rajabipour B, Mojtahedi A, Abdollahi M (2005). Effects of pentoxifylline on oxidative stress and levels of EGF and NO in blood of diabetic type-2 patients; a randomized, double-blind placebo-controlled clinical trial. Biomed Pharmacother.

[40] Arias-Diaz J, Vara E, Garcia C, Torres-Melero J, Rodriguez JM, Balibrea JL (1994). Pentoxifylline partially reverts the effect of tumor necrosis factor on human islets. Transplant Proc.

[41] Satapathy SK, Garg S, Chauhan R, Sakhuja P, Malhotra V, Sharma BC (2004). Beneficial effects of tumor necrosis factor-alpha inhibition by pentoxifylline on clinical, biochemical, and metabolic parameters of patients with nonalcoholic steatohepatitis. Am J Gastroenterol.

[42] Sharma BC, Kumar A, Garg V, Reddy RS, Sakhuja P, Sarin SK (2012). A Randomized Controlled Trial Comparing Efficacy of Pentoxifylline and Pioglitazone on Metabolic Factors and Liver Histology in Patients with Non-alcoholic Steatohepatitis. J Clin Exp Hepatol.

[43] Zein CO, Yerian LM, Gogate P, Lopez R, Kirwan JP, Feldstein AE (2011). Pentoxifylline improves nonalcoholic steatohepatitis: a randomized placebo-controlled trial. Hepatology.

